# Perfusion and Oxygenation of Random Advancement Skin Flaps Depend More on the Length and Thickness of the Flap Than on the Width to Length Ratio

**Published:** 2016-02-19

**Authors:** Khashayar Memarzadeh, Rafi Sheikh, Jonas Blohmé, Christian Torbrand, Malin Malmsjö

**Affiliations:** ^a^Department of Ophthalmology, Clinical Sciences, Lund University and Skåne University Hospital, Sweden; ^b^Department of Urology, Clinical Sciences, Lund University and Skåne University Hospital, Sweden

**Keywords:** perfusion, oxygenation, random flaps, experimental, blood flow

## Abstract

**Objective:** The aim was to investigate the relationship between the dimensions (length, width, and thickness) of random advancement skin flaps and retained tissue perfusion and oxygenation. **Methods:** Flaps were raised on the flanks of pigs. The flaps were either 0.5 or 1.0 cm wide, thin (dissected halfway through the subcutaneous tissue) or thick (dissected down to the muscle fascia). Tissue perfusion was measured by laser Doppler velocimetry, and tissue oxygenation (pO_2_) was measured using a Licox system, every 0.5 cm along the flaps’ length. Tissue temperature was visualized by high-resolution infrared camera. **Results:** Perfusion and oxygenation decreased gradually from the base to the tip of the flap, reaching approximately 40% of presurgical values (2.0 cm) and approximately 20% (2.5 cm) from the base of the flap. There was virtually no blood flow, nor oxygen tension, 3.0 cm from the base of the flap. The width to length ratio of the flap did not determine blood flow or oxygenation, being approximately 30% in a 0.5 cm wide and 2 cm long flap, and 0% in a 1.0 cm wide and 4 cm long flap, both with a width to length ratio of 1:4. Blood flow and oxygenation were preserved to a greater extent in the thick flaps (∼40%) than in the thin flaps (∼20%), in a 0.5 cm wide and 2 cm long flap. **Conclusions:** The dissection of a random advancement flap results in hypoperfusion and oxygenation that cannot be predicted by the width to length ratio but depend on the length and thickness of the flap.

Skin defects can be closed by wound healing by secondary intention, free skin grafts, or skin flaps. Skin flaps may be preferred since they maintain their original color and texture, undergo less contraction, and have their own blood supply. Clinical rules of thumb have emerged that govern the design of flaps, and the viable length of a flap is thought to depend on the width of its pedicle. The actual role of the length, width, and thickness of a random flap in its survival is still not clear.

In a textbook of plastic surgery from 1920, Sir Harold Gillies^1^ concluded that a flap should not be longer than the width of its base. In 1970, Milton^2^ studied the surviving area of rectangular flaps of different sizes in pigs. The surviving area of the flaps was found to have a constant ratio to the base of the flaps, and it was concluded that a constant area to base ratio was required. Work by others confirmed the clinical concept that the surviving length of a flap was dictated by its width, with the important proviso that there is an upper limit on the surviving length that cannot be increased by increasing the width of the base.^3^ Daniel and Williams^4^ studied random advancement flaps and found that an increase in width did not result in an increased length of survival. Stranc et al^5^ monitored rat dorsal skin flaps with near-infrared (IR) spectroscopy and reported that at a distance greater than 2 cm from the vascular pedicle, the tissue remained hypoxic.

Many of the previous studies on random advancement skin flaps have been conducted using experimental flap models in even smaller animals and rodents.^6^ The pig is considered to be a suitable animal for the study of full-thickness wounds, as the epidermis, dermis, and subcutaneous fat resemble those of humans.^7^^,^^8^ The blood supply to the pig flank consists of rows of segmental vessels, each vascular bundle containing a central artery with 2 concomitant venae. The skin is adherent to the deep fascia.^9^ When the skin flaps are dissected superficially to the panniculus carnosus, they will be of random pattern.^10^

Few studies have been conducted to investigate the combined role of the length, width, and thickness in preserving perfusion and tissue oxygenation in a random advancement flap, using large animals and modern techniques. The present study was conducted to investigate the relationship between the length, width, and thickness of the flap in an experimental porcine model of random advancement skin flaps. The microvascular blood flow was measured using invasive laser Doppler velocimetry, the tissue oxygenation was measured with invasive Licox probes, and tissue temperature was visualized with a high-resolution IR camera during surgery.

## METHODS

### Animals and anesthesia

Twelve pigs with a body weight of 70 kg were fasted overnight but with free access to water. An intramuscular injection of xylazine (Rompun vet. 20 mg/mL; Bayer AG, Leverkusen, Germany; 2 mg/kg) mixed with ketamine (Ketaminol vet. 100 mg/mL; Farmaceutici Gellini S.p.A, Aprilia, Italy; 20 mg/kg) was used for premedication. Anesthesia was then induced with intravenous sodium thiopental (Pentothal; Abbot Scandinavia, Stockholm, Sweden; 4 mg/kg) and fentanyl (Leptanal; Lilly, France; 2 μg/kg) and maintained with a continuous infusion of fentanyl in Ringer's acetate (3.5 µg/kg per hour) in combination with sodium thiopental (∼2.5 mg/kg). The animals were orally intubated with cuffed endotracheal tubes. Mechanical ventilation was established with a Siemens-Elema ventilator (Siemens-Elema AB, Solna, Sweden) in the volume-controlled mode (35% oxygen). The ventilation settings were identical for all animals: respiratory rate 15 breaths/min and minute ventilation 12 L/min. A positive end-expiratory pressure of 5 cm H_2_O was applied. A Foley catheter was inserted into the urinary bladder through a suprapubic cystostomy.

### Surgical procedure and laser Doppler and oxygen measurements

**Figure 1:**
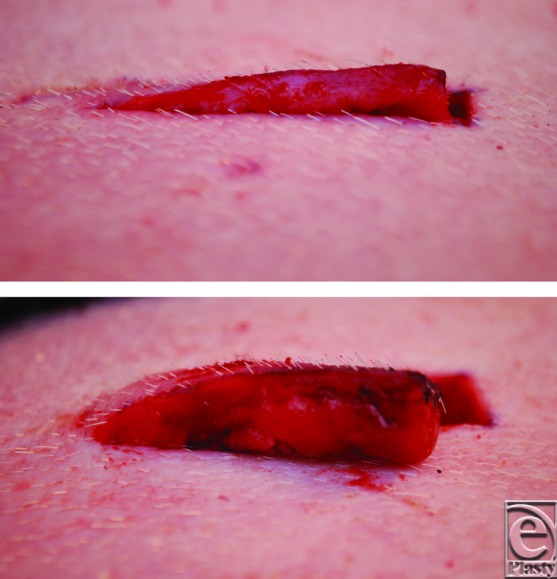
Photograph of the flaps dissected on the pig's flank (width: 1.0 cm; length: 4 cm). The flaps were either thin (top) or thick (bottom).

Rectangular random advancement flaps of different dimensions were dissected on each pig's flank (Fig 1). Short-term experiments were performed to study the immediate effects of surgery. For these experiments, blood flow and oxygenation were measured when blood flow and pO_2_ equilibrium had been observed for 1 hour. Long-term experiments were performed to investigate the role of vasospasm. For these experiments, blood flow and oxygenation were measured both after 1 and 8 hours. The flaps for the short-term experiments were made either 0.5 or 1.0 cm wide and either thin or thick. The thick flaps were dissected all the way through the subcutaneous tissue down to the muscle fascia, giving a thickness of about 0.8 to 0.9 cm, whereas thin flaps were only dissected halfway through the subcutaneous tissue, having a thickness of about 0.3 to 0.4 cm. Plastic cannulae were inserted every 0.5 cm along the length of the flaps, for laser Doppler and tissue oxygenation measurements. Separate flaps were dissected to perform long-term experiments. These flaps were made 1 cm wide and 4 cm long and thick. Cannulae for laser Doppler and Licox probes were placed at 1 and 3 cm from the flap base, and measurements were performed after 1 and 8 hours. After the blood flow and oxygenation measurements were complete, the blood supply to the pedicle of the flap was occluded using a 4-0 PDS II polydioxanone monofilament synthetic absorbable ligature. This procedure was performed to establish a reference value in which microvascular blood flow and pO_2_ were both assumed to be zero. In all experiments, a reference laser Doppler probe and a reference pO_2_ probe were inserted into adjacent tissue not subjected to surgery, in which the peripheral circulatory status of the pig was monitored.

### Laser Doppler velocimetry

Microvascular blood flow was measured by laser Doppler velocimetry using a 4-channel Perimed PF5010 unit (Perimed, Stockholm, Sweden). Laser Doppler velocimetry is a technique that quantifies the motion of red blood cells in a specific volume, and it has been applied extensively to measure blood flow in flaps during plastic surgery procedures.^11^ The present experiments were performed using filament probes. The filament probe (MT A500-0, straight microtip with slanted tip; Perimed, Stockholm, Sweden) was attached to a master probe (Probe 418), which was then connected to the laser Doppler equipment. The filament probe was 120 mm in length and had a 0.5-mm flexible microtip. The distal end of the tip has been polished to give it a slight angle to improve its optical properties. The probe was inserted into the tissue with a 22-G Venflon infusion cannula. The filament probe was inserted through a cannula, and the cannula was then withdrawn approximately 5 mm to expose the tip of the probe to the tissue. Recordings with baseline values between 100 and 10 perfusion units (PU) were analyzed in the study.

### Tissue oxygenation

Tissue oxygenation (pO2) was measured using a Licox CC1.SB system (Integra Neuroscience, Saint Priest, France), with a Clarke-type electrode inserted into the tissue using a 22-G Venflon infusion cannula. This probe is based on oxygen diffusion through an electrolyte chamber, which generates an electric current. The suitability and reliability of this probe have been demonstrated previously.^12^^-^14 Data were recorded continuously.

### IR imaging

Tissue temperature measurements were performed with a high-resolution IR camera (FLIR A655sc; FLIR Systems AB, Danderyd, Sweden). The IR camera was a focal plane array uncooled microbolometer with 640 × 480 pixel resolution and thermal sensitivity/NETD (noise equivalent temperature difference) less than 0.05°C at +30°C/50 mK. The IR camera was placed approximately 50 cm above the animal on a Manfrotto 244 Variable Friction Magic Arm that was mounted on a Manfrotto 190 series tripod. The software ThermaCAM Researcher Pro 2.10 from FLIR Systems was installed in a laptop personal computer and used for IR image capturing and postprocessing. The IR camera was connected to the laptop through the Ethernet interface using a 2-m shielded Ethernet cable. The camera's thermal sensitivity and resolution allow for measurement of temperature variation at the skin's surface. Change in temperature may be extrapolated to change in perfusion, with an increase in perfusion resulting in increased temperature, and vice versa. The change in skin temperature is believed to be proportional not only to changes in microcirculation but may also to other metabolic processes in the cells such as inflammatory responses and thermoregulatory enzymes. However, this limitation may be off less importance for interpretation of the present results since the focus was on temperature changes rather than actual temperature. Measurements were in the present study perfomed 1 hour after surgery. Imaging was performed on thin and thick flaps, and representative examples were presented.

### Calculations and statistics

Microvascular blood flow was expressed in PU and the tissue oxygenation in mmHg. The value when the flap pedicle had been occluded was set to 0%. The microvascular blood flow and tissue oxygenation recorded at increasing distance from the flap base are expressed as a percentage of the recordings in the flap base. Calculations and statistics were performed using GraphPad 6.0 software (San Diego, Calif). The results are presented as median values and ranges, using scatterplots or box-and-whisker plots in which the box is the 25th to 75th percentile, the whiskers are the minimum and maximum values (range), and the line is the median. Statistical analysis was performed using the Mann-Whitney test and Kruskal-Wallis test with the Dunn post hoc test for multiple comparisons. Significance was defined as *P* < .05 (*), *P* < .01 (**), *P* < .001 (***), and *P* > .05 (not significant, n.s.).

### Ethics

The experimental protocol for this study was approved by the Ethics Committee for Animal Research at Lund University, Sweden. All animals received humane care in compliance with the European Convention on Animal Care. The pigs were also used for other experiments, in which local effects of plastic or ophthalmological surgical techniques were examined.

## RESULTS

### The length of the flap

**Figure 2:**
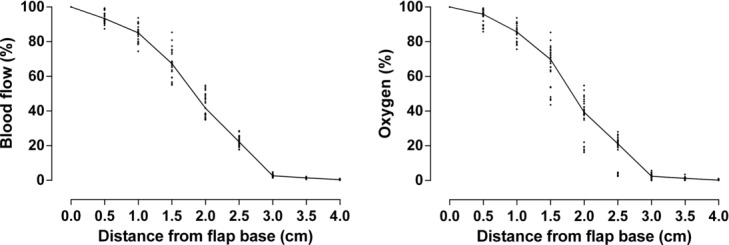
Blood flow (left) and oxygenation (right) at increasing distances from the flap base. The data are from both 0.5 and 1.0 cm wide thin and thick flaps (*n* = 24). The results are presented as scatterplots with a line through the median values. The results show the importance of the length of the flap in retaining blood flow and oxygenation.

Blood flow and the oxygenation of the tissue decreased gradually from the base to the tip of the flap. Blood flow and oxygenation were reduced to approximately 40% of presurgical values when measured 2.0 cm from the base and to approximately 20% when measured 2.5 cm from the base. There was virtually no blood flow, nor oxygen tension, 3.0 cm from the base. The length of the flap seemed to be of major importance in preserving blood flow and oxygenation to the tip of the flap. The results are shown in Figure 2.

**Figure 3:**
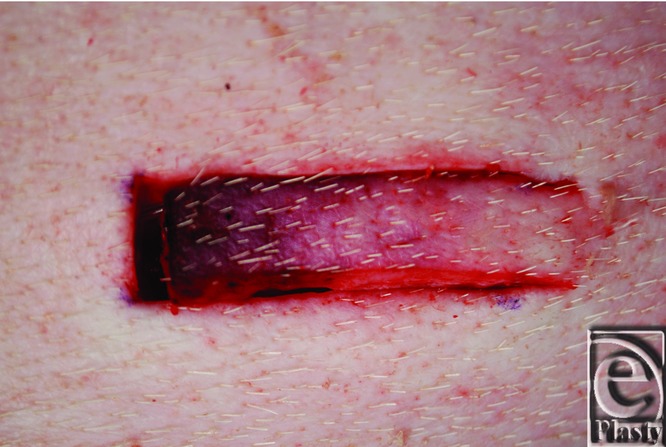
Random advancement skin flap (1 × 4 cm) on the pig flank, 1 hour after surgery, at the time of perfusion and oxygenation measurements. It can be seen that approximately the distal 2 cm of the flap is discolored.

Discoloring of the distal end of the flap developed progressively over time. One hour after dissection of the flap, at the time of the blood flow and oxygenation recordings, the discolored flap length was equivalent to the region devoid of perfusion or oxygenation. The discolored flap length was difficult to measure accurately and was therefore not determined or analyzed statistically. Figure 3 shows a representative photograph of a discolored flap.

### Width to length ratio

**Figure 4:**
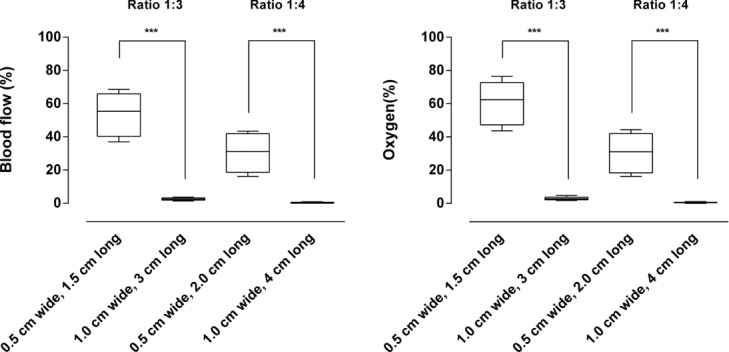
Blood flow (left) and oxygenation (right) in random advancement flaps of different dimensions, with width to length ratios of 1:3 and 1:4 (both thin and thick flaps, *n* = 12 for each box). Note that the width to length ratio of the flap does not determine the blood flow or oxygenation. ***P < .001.

The width to length ratio of the flap did not determine the blood flow or oxygenation. For example, in 2 different flaps, both with a width to length ratio of 1:4, about 30% of the blood flow was preserved 2 cm from the base in a 0.5 cm wide and 2.0 cm long flap while no blood flow was seen 4 cm from the base in a 1.0 cm wide and 4.0 cm long flap (Fig 4).

### Thick versus thin flaps

**Figure 5:**
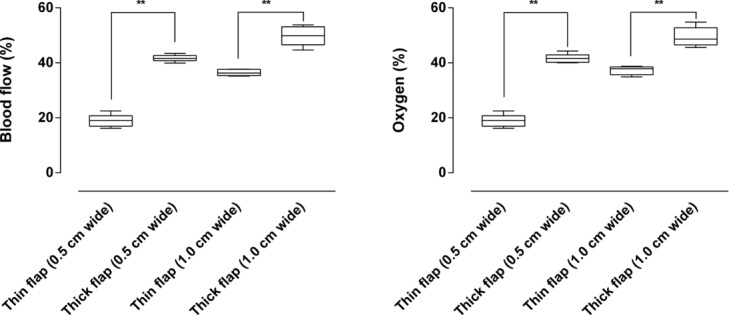
Blood flow (left) and oxygenation (right) in thin and thick flaps (*n* = 6 for each box), measured 2.0 cm from the flap base. Note that blood flow and oxygenation were preserved to a greater extent in thick than in thin flaps. **P < .01.

**Figure 6:**
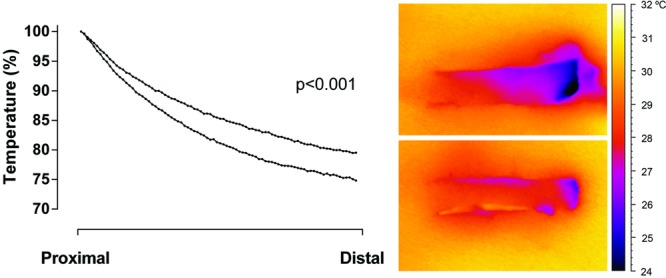
Thermographic images of 2 random advancement skin flap (1 × 4 cm) on the pig flank, 1 hour after surgery. The upper flap was made thin and the lower flap was made thick. Graph shows the drop in temperature along the length of the flaps (upper curve are thin flaps and lower curve are thick flaps), calculated as percent of a perfusion at a reference point lateral to the flap base (*n* = 10). Note the lower temperature seen as darkening of the distal end of the flap.

Blood flow and oxygenation were preserved to a greater extent in the thick flaps (∼40%) than in the thin flaps (∼20%) when measured 2.0 cm from the flap base, in a 0.5 cm wide flap. This difference seemed to be of slightly greater importance in the narrow flap (0.5 cm wide) than in the wider flap (1.0 cm wide). See Figure 5 for detailed results. Visualization using high-resolution IR camera showed decreased tissues temperature in the distal end of the flaps. The drop in temperature seemed more pronounced in thin flaps than in thick flaps, as shown in the graph and representative example in Figure 6.

### Long-term effects

The effects on blood flow persisted 8 hours after surgery. Immediately after surgery, the blood flow was 86 (26–183) PU, 1 cm from the flap base, and 4 (0–29) PU, 3 cm from the flap base. Eight hours later, the blood flow was 89 (28–210) PU, 1 cm from the flap base, and 7 (0–41) PU, 3 cm from the flap base (*P* = n.s.; *n* = 10), suggesting that surgical vasospasm does not play a major role in the flap hypoperfusion studied.

## DISCUSSION

In clinical practice, the design of random advancement flaps is largely determined by empirical rules and it is believed that these should be of constant width to length ratio.^1^^,^^2^ The results from the present study show that there is no linear relationship between the width to length ratio of the flap and perfusion or oxygenation. Instead, we found that the thickness and length of the flap are important for perfusion and oxygenation.

A thick flap, which was dissected all the way through the subcutaneous tissue down to the muscle fascia, showed better perfusion and oxygenation than a thin flap, which was only dissected halfway through the subcutaneous tissue. It is well known that the blood supply to random advancement skin flaps is derived from the dermal and subdermal vascular plexuses, and they have no specific vascular pedicle. The blood flow derives from the segmental artery that gives off branches that penetrate the muscle layers and run into the skin perpendicularly, each branch supplying a small area of skin. The reason that the blood flow is better preserved in a thick flap is probably because the vascular network connected to the pedicle and the flap is larger than in a thin flap.

Interestingly, the literature suggests that survival can be affected by the dimensions of the flap.^3^ This is supported by the results of the present study, in which blood flow and oxygenation were reduced to approximately 40% of the presurgical values when the flap was 2.0 cm long and to approximately 20% when it was 2.5 cm long. Virtually no blood flow was seen in the tip of the flap 3.0 cm from the base. The findings regarding blood flow and oxygenation were supported by the observation of discoloration of the tip of the flap 1 hour after dissection.

One limitation of the present study is that the majority of the measurements were performed only 1 hour after dissection of the flap. Vasospasm may occur around the flap pedicle,^6^ and the role of surgical vasospasm should be considered. We therefore monitored the blood flow in some of the flaps for 8 hours and found that the perfusion was similar to that 1 hour after surgery, suggesting that surgical vasospasm was not involved.

In conclusion, the results of the present study suggest that the degree of perfusion and oxygenation of a random advancement skin flap cannot be predicted by the width to length ratio. Tissue perfusion and oxygenation decreased gradually from the base to the tip of the flap, being approximately 40% of presurgical values 2.0 cm from the base, approximately 20% 2.5 cm from the base, and 0% 3.0 cm from the base of the flap. Dissecting a thick flap that extends deeper into the subcutaneous tissue resulted in greater perfusion and oxygenation than dissecting a thin flap. The length and thickness together may be important characteristics of the flap, but the width to length ratio appears to play a minor role.
